# Human Skin Odor Throughout Life: A Literature Review

**DOI:** 10.1111/jocd.71194

**Published:** 2026-09-15

**Authors:** Ghada Farouk Mohammed, Ahmed Kaid Alantary, Mohammed Saleh Al‐Dhubaibi, Saleh Salem Bahaj, Nada Sameh Aziz, Ahmed Mohammed Al‐Dhubaibi

**Affiliations:** ^1^ Department of Dermatology, Venereology, and Sexology, Faculty of Medicine Suez Canal University Ismailia Egypt; ^2^ Department of Basic Health Sciences, Physiology, College of Applied Medical Sciences Qassim University Buraydah Saudi Arabia; ^3^ Department of Dermatology, Dawadmi College of Medicine Shaqra University Shaqra Saudi Arabia; ^4^ Department of Microbiology and Immunology, Faculty of Medicine and Health Sciences Sana'a University Sanaa Yemen; ^5^ Faculty of Medicine Suez Canal University Ismailia Egypt; ^6^ College of Medicine Marmara University Istanbul Turkey

**Keywords:** 2‐nonenal, age, analytical methods, microbiome, narrative review, sex differences, skin odor, translational potential, volatile organic compounds, volatilome

## Abstract

**Background:**

Human skin continuously releases volatile organic compounds (VOCs) that constitute the skin volatilome, a dynamic chemical signature reflecting genetic, hormonal, microbial, and environmental influences.

**Objective:**

To provide a comprehensive, critically appraised synthesis of current knowledge on the human skin volatilome across the lifespan, emphasizing age‐related dynamics, sex‐specific profiles, microbiome contributions, analytical methodologies, and translational applications, while systematically evaluating evidence quality and identifying knowledge gaps.

**Methods:**

We conducted a narrative review with systematic search methodology. A systematic search of PubMed/MEDLINE, Scopus, and Web of Science was performed from database inception to May 2026, following a pre‐defined protocol. Of 487 initially identified records, 55 studies met the inclusion criteria following systematic screening, full‐text assessment, and reference list review. Quality appraisal was performed using five predefined criteria (sample size adequacy, analytical validation, control for confounding, study design appropriateness, and reporting completeness), with studies graded as High (≥ 4 criteria met), Moderate (3 criteria met), or Low (≤ 2 criteria met). Data were synthesized thematically across six domains: chemical composition, lifespan dynamics, sex differences, microbiome determinants, analytical methodologies, and translational applications.

**Results:**

Age‐dependent transformations are well‐documented: infant odor is dominated by acidic VOCs; puberty introduces qualitative changes via apocrine gland activation; reproductive‐age profiles exhibit consistent sex‐specific patterns; geriatric odor is characterized by 2‐nonenal emergence. Quality appraisal revealed that 22 studies (38%) were rated high quality, 24 (41%) moderate, and 12 (21%) low, with small sample sizes (median *n* = 35) and lack of analytical validation being the primary limitations. The skin microbiome, particularly *Corynebacterium*, *Cutibacterium*, and *Staphylococcus*, significantly modulates odor profiles.

**Conclusions:**

The skin volatilome reflects age, sex, microbiome, and health status. While VOC analysis offers promise as a noninvasive biosensing approach, methodological heterogeneity, limited longitudinal data, and incomplete mechanistic understanding constrain translation.

**Abbreviations**
3M2H: 3‐methyl‐2‐hexenoic acid
GC×GC‐TOFMS: comprehensive two‐dimensional gas chromatography‐time‐of‐flight mass spectrometry
GC‐MS: gas chromatography–mass spectrometry
PTR‐MS: proton transfer reaction‐mass spectrometry
SANRA: Scale for the Assessment of Narrative Review Articles
SPME: solid‐phase microextraction
TD: thermal desorption
VOC: volatile organic compound

## 1 Introduction

The skin volatilome comprises hundreds of compounds spanning multiple chemical classes, with concentrations varying by anatomical site, physiological state, and individual characteristics [1]. This chemical interface between the body and environment represents a unique window into human biology, reflecting both endogenous metabolism and external influences [2]. The study of the skin volatilome lies at the intersection of chemical ecology, analytical chemistry, microbiology, and clinical medicine, offering unique insights into human biology and disease [3]. Contemporary advances in analytical chemistry, microbial ecology, and systems biology have transformed skin odor research from descriptive curiosity to quantifiable biology with diagnostic and therapeutic applications [4, 5].

These molecules originate from multiple sources: eccrine and apocrine gland secretions, sebaceous products, epidermal lipid peroxidation, microbial metabolism of host‐derived substrates, and systemic metabolic byproducts that diffuse from the bloodstream through the skin [6]. The relative contribution of each source varies by body region, with apocrine glands predominantly influencing axillary odor and epidermal lipid peroxidation contributing to whole‐body emissions. Characteristic axillary odor is primarily attributed to volatile fatty acids, particularly 3‐methyl‐2‐hexenoic acid (3M2H), and steroid derivatives including androstenone and androstanol [7]. The evolutionary significance of skin odor manifests in intraspecies communication, mate selection, kin recognition, and disease detection [8]. Studies demonstrate that human infants recognize mothers by odor and that mothers identify their own infant's odor. Preliminary evidence suggests individuals may identify family members through olfactory cues, though this requires further validation and may be limited to specific kinship relationships [9, 10].

Despite substantial progress, significant knowledge gaps persist. The systematic characterization of skin odor across the complete human lifespan remains incomplete, with a notable absence of comprehensive longitudinal investigations [11]. Sex differences, while documented, have unclear contributions from genetic, hormonal, microbial, and behavioral factors [12]. The skin microbiome is increasingly recognized as a primary odor determinant, yet precise metabolic pathways remain incompletely characterized [13]. Diagnostic applications show promise, but most studies are small, cross‐sectional, and conducted under controlled laboratory conditions that may not reflect clinical reality, with inadequate control for confounding factors and limited independent validation [14]. Considerable methodological heterogeneity in sampling techniques, analytical instrumentation, and statistical approaches limits direct comparisons and meta‐analyses.

The existing literature is fragmented across scientific disciplines, with no comprehensive synthesis integrating chemical ecology, analytical chemistry, microbiology, endocrinology, clinical medicine, and forensic science within a lifespan framework. Several recent reviews have addressed specific aspects [15, 16, 17], but none have provided a critically appraised, integrated lifespan perspective that systematically evaluates evidence quality, reconciles contradictory findings, and proposes a unifying conceptual framework. As highlighted by Di Cicco et al. [17], axillary odor variation arises from a complex interplay of intrinsic factors (genetics, age, and hormones) and extrinsic factors (hygiene, diet, and environment), underscoring the multifactorial nature of human body odor.

Building on recent reviews that have addressed specific aspects of skin odor [15, 16, 17], this review provides a comprehensive, critically appraised, integrated lifespan synthesis that systematically evaluates evidence quality, reconciles contradictory findings, and proposes a unifying conceptual framework across disciplinary boundaries. Specifically, this review addresses this gap by providing: (1) a comprehensive, critically appraised synthesis of evidence on skin volatilome composition and age‐related dynamics from infancy to geriatric age; (2) systematic evaluation of sex‐specific patterns and their biological underpinnings; (3) critical assessment of the skin microbiome's role in modulating odor signatures, including metabolic pathways and evidence quality; (4) evaluation of clinical applications and diagnostic potential with explicit acknowledgment of limitations; (5) methodological critique and standardization recommendations; and (6) a proposed conceptual framework integrating determinants across biological levels. Through this synthesis, we identify robust findings, highlight knowledge gaps, assess evidence quality, and propose research priorities to advance understanding of the human skin volatilome and its translational potential.

## 2 Methods

### 2.1 Study Design and Rationale

This review was conducted as a narrative review with systematic search methodology, a hybrid approach that combines the rigorous, transparent search, screening, and data extraction methods of systematic reviews with the integrative, critical synthesis characteristic of high‐quality narrative reviews [18, 19]. This methodology is well‐suited for synthesizing evidence across heterogeneous disciplines (chemical ecology, analytical chemistry, microbiology, endocrinology, clinical medicine, and forensic science) where the evidence base comprises diverse study designs that preclude quantitative meta‐analysis.

The review was structured to address six thematic areas: chemical composition, lifespan dynamics, sex differences, microbiome interactions, analytical methodologies, and translational applications. The narrative approach enabled flexible integration of findings from original research articles while maintaining scientific rigor and transparency through systematic search, explicit eligibility criteria, independent screening, and systematic quality appraisal.

The choice of this methodology was driven by: (a) the heterogeneous nature of the evidence base (cross‐sectional, cohort, case–control, experimental studies); (b) the need for critical synthesis across multiple disciplines; (c) the importance of contextualizing findings within broader biological frameworks; and (d) the goal of providing evidence‐based recommendations for clinical translation and future research. Quality appraisal was performed using criteria adapted from the Scale for the Assessment of Narrative Review Articles (SANRA) [18], supplemented with field‐specific methodological criteria.

The review protocol, including the research questions, search strategy, eligibility criteria, and quality appraisal framework, was developed a priori by all authors and is available upon request from the corresponding author. Protocol registration was not performed as this is not required for narrative reviews [18, 19].

### 2.2 Literature Search Strategy

We performed a systematic search of three electronic databases: PubMed/MEDLINE, Scopus, and Web of Science. The search period spanned from database inception to May 15, 2026. Searches were limited to English‐language publications.

The search strategy combined terms related to skin volatilome, biological determinants (microbiome), lifespan/age, sex/gender, and analytical methods using Boolean operators (AND, OR). Complete search strategies for all three databases are provided in Appendix S1.

Reference lists of identified articles were manually screened for additional relevant publications. Citation tracking was performed using Google Scholar to identify studies that had cited key publications. All retrieved records were exported to EndNote X9 for duplicate removal and screening.

### 2.3 Eligibility Criteria

#### 2.3.1 Inclusion Criteria

Studies were included if they met the following criteria:

- Population: Human subjects of any age, sex, ethnicity, or health status
- Intervention/Exposure: Measurement of skin VOC profiles or odor assessment
- Comparators: Age groups, sex, health status, or other relevant comparisons
- Outcomes: VOC identification, quantification, or characterization; odor perception; clinical correlations
- Study design: Original research (cross‐sectional, cohort, case–control, experimental) or reviews
- Analytical validation: Use of validated analytical techniques (GC‐MS, GC×GC‐TOFMS, PTR‐MS, SIFT‐MS, or equivalent) for VOC confirmation
- Language: English
- Publication date: 1981–2026 (with landmark pre‐1981 studies included for historical context)

Foundational landmark studies published before 1981 were included, which established key principles in the field. Studies with adequate methodological detail to permit evaluation of findings were prioritized. Reviews and secondary sources were included only for contextual framing and reference identification, not as primary evidence for VOC composition or clinical associations.

#### 2.3.2 Exclusion Criteria

Studies were excluded if they met any of the following criteria:

- Publication type: Case reports, editorials, commentaries, opinion pieces, and conference abstracts
- Population: Animal studies, in vitro studies without human relevance
- Focus: Fragrance or cosmetic product evaluation without natural odor assessment; breath or urinary odor without skin component; animal studies (except where used for mechanistic contextualization in Discussion)
- Methodology: Studies using only subjective odor assessment without analytical confirmation
- Quality: Insufficient methodological detail to permit evaluation; overlapping datasets (only the most comprehensive report included)
- Language: Non‐English publications

### 2.4 Study Selection

Study selection followed a structured, multi‐stage process:

1. Identification: Retrieved records were imported into reference management software, where duplicate records were identified and removed.
2. Screening: Two independent reviewers performed title and abstract screening against the pre‐established eligibility criteria. Records were classified as follows: include (clearly meeting criteria), exclude (clearly not meeting criteria), or uncertain (insufficient information).
3. Eligibility: Full texts of all included and uncertain records were obtained for detailed assessment. The same two reviewers independently evaluated full‐text articles against the eligibility criteria, with reasons for exclusion systematically documented.
4. Inclusion: Disagreements between reviewers were resolved through discussion and consensus, with a third reviewer (AKAA) consulted to adjudicate persistent disagreements

**FIGURE 1:**
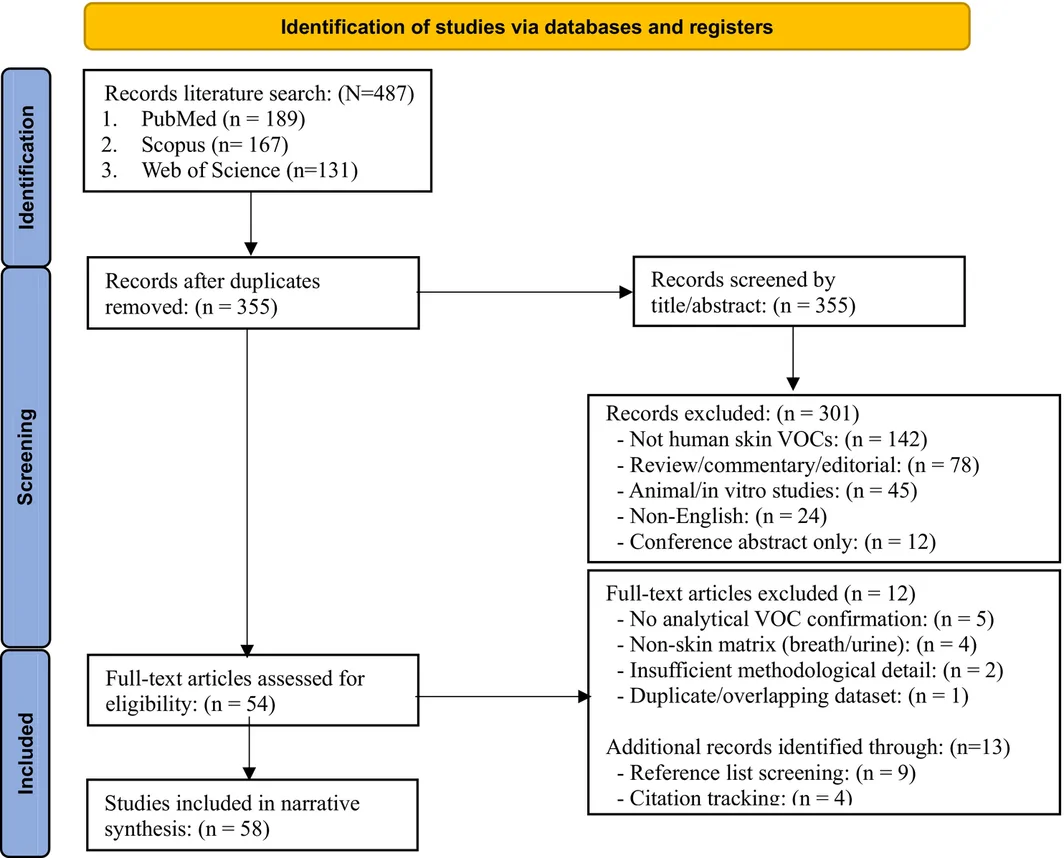
Flow diagram of study selection process.

Our search yielded 487 records. After duplicate removal (*n* = 132), title/abstract screening (*n* = 355; 301 excluded), and full‐text assessment (*n* = 54; 12 excluded), 42 studies met the inclusion criteria. Manual reference screening and citation tracking identified an additional 16 studies, resulting in a final total of 55 included studies. The study selection process is summarized in a flow diagram (Figure 1), which details the number of records identified, screened, assessed for eligibility, reasons for exclusion, and studies included in the review.

### 2.5 Data Extraction

Data extraction was performed using a standardized, pilot‐tested data extraction form developed specifically for this review. Two independent reviewers extracted data from each included study, with discrepancies resolved through consensus discussion. Extracted variables were organized to capture comprehensive information:

- Study characteristics: Author(s), year, country, study design, setting, sample size, and participant characteristics
- Participant demographics: Age, sex, ethnicity, BMI, health status, skin type, and body region sampled
- Methodological details: Sampling technique, analytical instrumentation, compounds identified, and quantification method
- Odor composition: VOC identities, relative abundances, chemical classes, age‐related patterns, and sex‐specific compounds
- Microbiome data: Sampling method, microbial identification approach, species composition, and correlation with VOCs
- Clinical data: Health conditions, disease states, treatment status, and clinical outcomes
- Key findings: Main results, age comparisons, sex differences, microbiome associations, and clinical correlations

Extracted evidence was categorized according to:

- Strength of evidence: Strong (consistent findings across ≥ 3 high‐quality studies), Moderate (consistent but limited studies), and Weak (inconsistent or single studies)
- Study design: Cross‐sectional, prospective cohort, case–control, randomized controlled trial, longitudinal, experimental
- Quality grade: High, Moderate, or Low based on the narrative review quality criteria described below

### 2.6 Quality Appraisal for Narrative Review

The SANRA criteria were adapted for this narrative review as a quality assessment framework, supplemented with field‐specific methodological criteria to address the unique requirements of skin VOC studies. This approach aligns with established precedents for quality assessment in narrative reviews [18]. The quality assessment was performed to contextualize evidence strength and identify methodological limitations, not as an inclusion/exclusion criterion.

Quality appraisal was performed using criteria adapted for narrative reviews in the absence of a validated tool specific to skin VOC studies [18]. Two independent reviewers assessed each included study based on five key methodological aspects, each scored as present (1) or absent/insufficient (0): following key methodological aspects:

1. Sample size adequacy: For comparative studies, ≥ 20 participants per group; for descriptive studies, ≥ 50 participants total, based on power calculations for detecting moderate effect sizes (Cohen's *d* ≥ 0.5) with *α* = 0.05, *β* = 0.
2. Analytical validation: VOC confirmation using authentic standards, or robust spectral matching with ≥ 90% match and retention index verification.
3. Control for confounding: Adjustment for at least three of the following: age, sex, diet, hygiene, medications, or environmental exposures.
4. Study design appropriateness: Design matched to research question with appropriate control groups or within‐subject controls.
5. Reporting completeness: Sufficient methodological detail to permit replication of sampling, analysis, and data processing steps.

Studies scoring 4–5 were graded High, 3 were graded Moderate, and 0–2 were graded Low. This scoring system was pilot‐evaluated on five studies to ensure inter‐rater reliability (Cohen's *κ* > 0.80).

### 2.7 Data Synthesis

A thematic synthesis approach was employed to organize findings across the identified literature. This method was selected due to the anticipated heterogeneity in study designs, methodologies, and outcome measures. Key themes were developed iteratively through careful examination of the literature and refined to reflect the major domains of skin odor research.

The thematic analysis framework comprised six major categories:

1. Chemical composition: VOC classes, compounds, regional variations, and baseline profiles
2. Lifespan dynamics: Odor characteristics across infancy, childhood, puberty, reproductive age, and geriatric periods.
3. Sex differences: Male–female VOC differences, hormonal influences, and pubertal divergence
4. Microbiome determinants: Microbiome composition‐odor associations, specific microbial contributions, and dysbiosis effects
5. Analytical methodologies: Sampling techniques, analytical platforms, and data analysis approaches
6. Translational and diagnostic potential: Disease associations, diagnostic potential, and forensic applications

Evidence integration was performed by systematically comparing findings across studies to identify:

- Consistent patterns: Findings replicated across multiple studies with supporting evidence
- Inconsistent results: Conflicting findings requiring investigation and discussion
- Gaps: Topics inadequately addressed in the literature
- Emerging trends: Promising patterns requiring further validation

The narrative synthesis is presented with supporting data organized in summary tables and structured by thematic categories. Quantitative data (where available from ≥ 3 studies) were summarized using range, mean, or weighted mean. Effect sizes and confidence intervals were reported where available.

## 3 Results

### 3.1 Chemical Composition of Human Skin Odor

The skin volatilome comprises a complex mixture of VOCs spanning multiple chemical classes. The most comprehensively studied body region is the axilla, followed by the hand, forearm, and whole‐body emissions [1, 15, 16]. Across 55 included studies, the most frequently detected VOC classes include short‐chain fatty acids, aldehydes, ketones, alcohols, esters, hydrocarbons, and sulfur‐ and nitrogen‐containing compounds [15, 20, 21, 22].

The 55 included studies comprised 25 examining chemical composition, 18 focusing on lifespan dynamics, 15 addressing sex differences, 14 investigating microbiome determinants, 20 evaluating analytical methodologies, and 12 exploring clinical applications, with substantial overlap between thematic areas.

Table 1 summarizes the major chemical classes, representative compounds, body region associations, detection frequency across studies, and evidence quality. Detection frequency was calculated as the percentage of studies (among those analyzing the relevant body region) reporting the compound. Only compounds detected in ≥ 3 independent studies are included.

**TABLE 1:** Major chemical classes of human skin VOCs.

Chemical class	Representative compounds	Body region	Frequency of detection	Evidence type	Key references
Fatty acids	Acetic acid, propionic acid, isovaleric acid, 3M2H, (E)‐3M2H	Axilla, whole body	High (80%–100%)	Primary (Human Skin)	[15, 20, 21, 23]
Aldehydes	Hexanal, heptanal, octanal, nonanal, decanal, (E)‐2‐nonenal, (E, E)‐2,4‐nonadienal	Whole body, skin surface	High (70%–95%)	Primary (Human Skin)	[1, 15, 24, 25]
Ketones	6‐Methyl‐5‐hepten‐2‐one, 2‐heptanone, 2‐nonanone, geranylacetone	Axilla, whole body	High (65%–90%)	Primary (Human Skin)	[15, 24, 26]
Alcohols	1‐Hexanol, 1‐octen‐3‐ol, 2‐ethyl‐1‐hexanol, phenylethyl alcohol	Axilla, skin	Moderate‐High (50%–75%)	Primary (Human Skin)	[1, 15, 16]
Esters	Ethyl acetate, isopropyl myristate, fatty acid esters	Skin surface	Moderate (40%–65%)	Primary (Human Skin)	[1, 15, 16]
Hydrocarbons	Alkanes (C8‐C30), alkenes, terpenes	Whole body	Moderate‐High (55%–80%)	Primary (Human Skin)	[15, 16, 24]
Sulfur Compounds	Methanethiol, dimethyl disulfide, thioesters	Axilla	Moderate (35%–60%)	Primary (Human Skin)	[16, 21, 27]
Nitrogen Compounds	Indole, skatole, amines	Axilla, feet	Moderate (30%–55%)	Primary (Human Skin)	[1, 15, 16]
Steroids	5α‐Androst‐16‐en‐3‐one (androstenone), 5α‐androst‐16‐en‐3α‐ol (androstanol)	Axilla	Moderate (40%–60%)	Primary (Human Skin)	[7, 15, 20, 21]

*Note:* Evidence type: Primary (Human Skin) = original research measuring VOCs from human skin, meeting inclusion criteria. All compounds listed in this table are from primary human skin VOC studies.

Fatty acids, particularly short‐chain and branched‐chain variants, constitute the most abundant class of skin VOCs, with 3M2H consistently identified as the characteristic axillary odorant across diverse populations [20, 21, 23]. The compound (E)‐ 3M2H has also been identified as a key odor‐active compound contributing to axillary sweat odor [27]. Aldehydes, including hexanal, heptanal, octanal, nonanal, and the geriatric‐associated (E)‐2‐nonenal, are abundant in whole‐body emissions and skin surface collections [25, 26]. Ketones such as 6‐methyl‐5‐hepten‐2‐one, 2‐heptanone, and 2‐nonanone are commonly detected in axillary and whole‐body samples [1].

**FIGURE 2:**
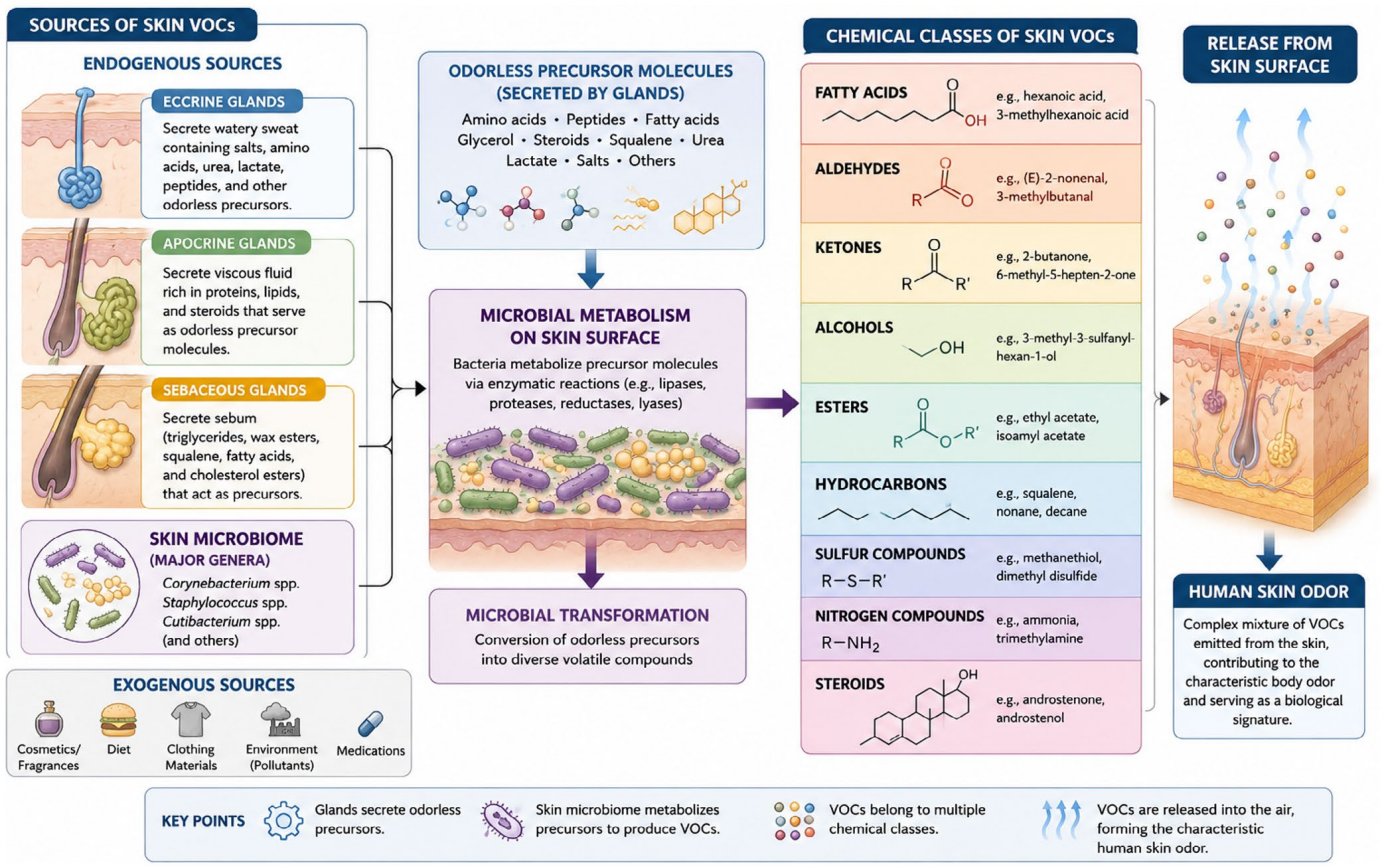
Sources and chemical classes of human skin volatile organic compounds. Endogenous sources: Eccrine glands (water, salts, and acids), Apocrine glands (steroid precursors and proteins), and Sebaceous glands (lipids and fatty acids). Exogenous sources: Skin microbiome (*Corynebacterium*, *Staphylococcus*, and *Cutibacterium*), Environmental exposures. Major VOC classes: Fatty acids, Aldehydes, Ketones, Alcohols, Esters, Hydrocarbons, Sulfur compounds, Nitrogen compounds, and Steroids. Arrows indicating microbial conversion of odorless precursors to volatile odorants.

Figure 2 provides a visual overview of endogenous sources (eccrine, apocrine, and sebaceous glands) and exogenous sources (skin microbiome), along with the major chemical classes of VOCs released from the skin surface.

### 3.2 Axillary Odor Chemistry

The axillary region has received the most intensive investigation due to its high density of apocrine glands and characteristic odor [7, 20, 27, 28]. Apocrine gland secretions contain odorless precursor compounds that are converted to volatile odorants through bacterial action [23]. A specific bacterial aminoacylase in *Corynebacterium* species cleaves these odorant precursors, releasing 3M2H and other odor‐active compounds [23]. Di Cicco et al. [17] comprehensively reviewed the intrinsic and extrinsic factors, including genetics, age, hormones, hygiene, diet, and environment, which contribute to axillary odor variation.

Troccaz et al. [27] identified 3‐methyl‐3‐sulfanylhexan‐1‐ol as a major descriptor for the human axilla‐sweat odor profile, demonstrating the contribution of sulfur‐containing compounds to axillary malodor. Subsequent studies have characterized the full spectrum of volatile compounds in axillary secretions, identifying over 40 individual VOCs that collectively contribute to the characteristic axillary odor [1, 20].

**FIGURE 3:**
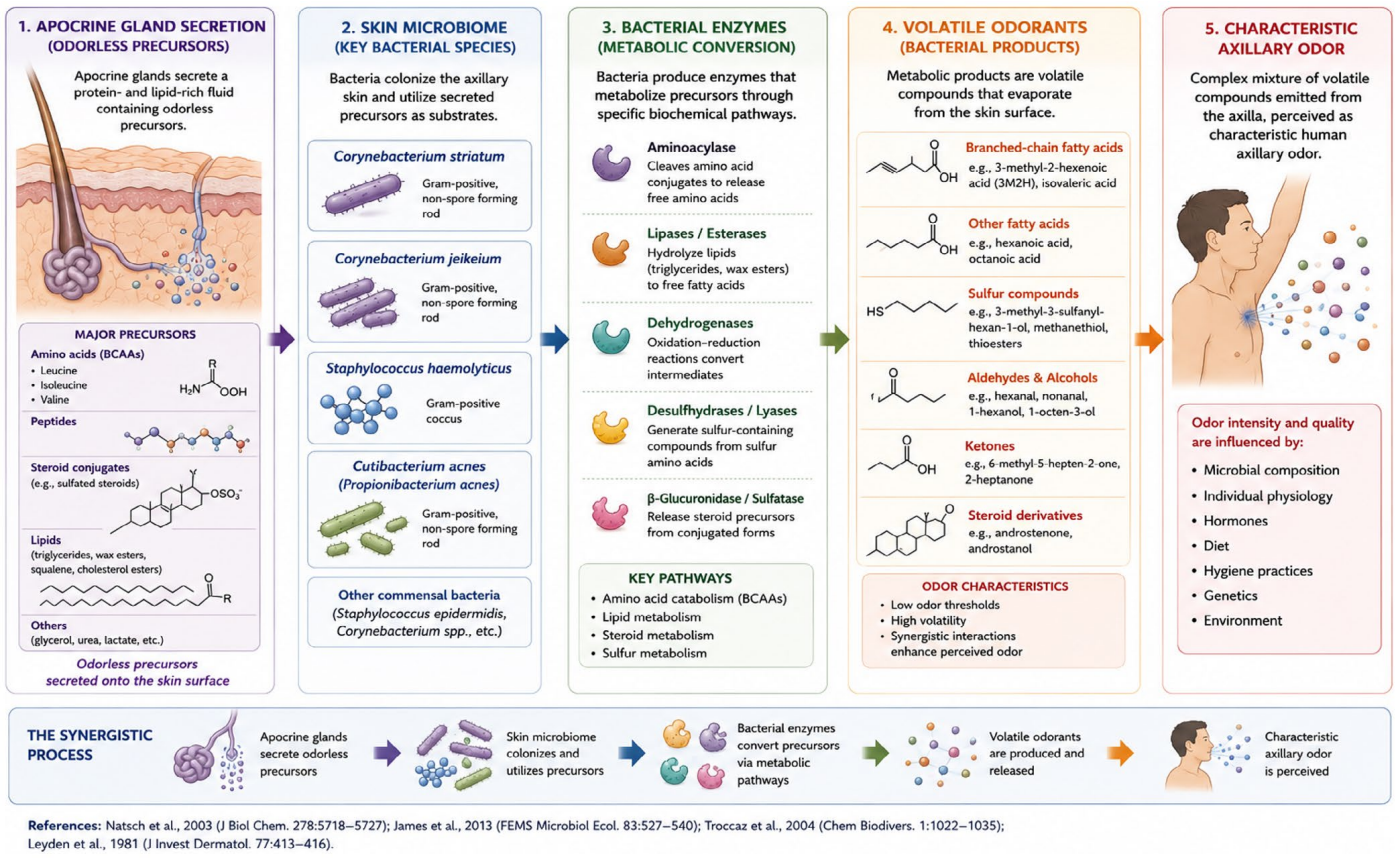
Axillary odor formation: Synergistic role of apocrine glands and skin microbiome. Apocrine gland secretion of odorless precursors (amino acid conjugates, steroid conjugates). Bacterial enzymatic pathways: Aminoacylase (C*orynebacterium*), Lipase (*Staphylococcus*), Steroid dehydrogenase (*Cutibacterium*). Release of volatile odorants: 3M2H, isovaleric acid, androstenone, androstanol. Key bacterial species: *Corynebacterium striatum* , *C. jeikeium* , *Staphylococcus haemolyticus* , *Cutibacterium acnes*.

Figure 3 illustrates the biochemical pathway by which odorless apocrine gland secretions are converted to volatile odorants through bacterial metabolism. This figure highlights the synergistic role of apocrine glands and skin microbiome in axillary odor formation, depicting key bacterial species ( *Corynebacterium striatum* , *Corynebacterium jeikeium* , *Staphylococcus haemolyticus* , and *Cutibacterium acnes*) and their enzymatic pathways.

### 3.3 Regional Variation

The skin volatilome varies considerably across anatomical sites, reflecting regional differences in glandular density, microbiome composition, and environmental exposure [1, 16]. Finnegan et al. [11] demonstrated that skin volatile profiles show strong predictive ability for age (explained variance of 68%), with significant correlations between specific VOCs and participant age, and identified gender influences on VOC profiles. Whole‐body emissions are characterized by a broader range of aldehydes, ketones, and hydrocarbons compared to axillary secretions, reflecting contributions from eccrine glands, sebaceous glands, and epidermal lipid peroxidation [29].

The hand and forearm regions have been extensively studied due to their accessibility and suitability for non‐invasive sampling. Studies of these regions have identified aldehydes, particularly hexanal, heptanal, octanal, and nonanal, as major constituents, with concentrations varying by age, sex, and health status [22]. Finnegan et al. [11] demonstrated that skin volatile profiles from the forearm show strong predictive ability for age (explained variance of 68%), with significant correlations between specific VOCs and participant age, and identified gender influences on VOC profiles.

Quantitatively, whole‐body VOC emission rates show considerable inter‐individual variation. Mochalski et al. [22] reported mean emission rates of 0.1–5.0 ng/cm^2^/h for aldehydes and 0.5–20.0 ng/cm^2^/h for fatty acids, with coefficients of variation typically exceeding 50%. This high variability underscores the importance of standardized sampling protocols and adequate sample sizes in comparative studies.

### 3.4 Lifespan Dynamics of Skin Odor

#### 3.4.1 Infant and Neonatal Odor (0–12 Months)

Studies on neonatal and infant odor reveal distinctive profiles that differ significantly from adult patterns [30]. Infant skin odor is characterized by a predominance of acidic VOCs, particularly acetic acid, propionic acid, and other short‐chain fatty acids, along with sweet, milky notes from γ‐ and δ‐lactones [1]. Notably, infant odors lack androgenic compounds such as androstenone and androstanol that are found in adult axillary odors [30].

**FIGURE 4:**
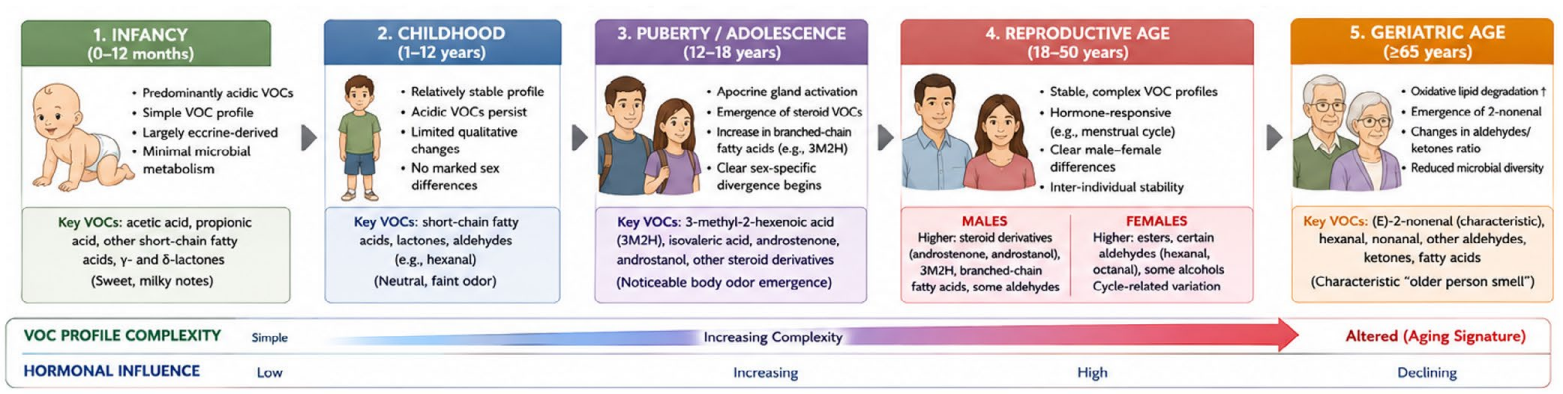
Lifespan trajectory of human skin odor. Evidence quality graded as High (≥ 3 high‐quality studies with consistent findings), Moderate (consistent findings but limited studies), or Low (inconsistent or single studies).

Prokop‐Prigge et al. [30] demonstrated that mothers can identify their own infant's body odor with 90% accuracy, supporting the biological significance of infant odor in maternal bonding. The infant profile appears to be primarily derived from eccrine gland secretions and vernix caseosa residues, with limited microbial metabolism compared to adults [1]. Quality assessment of the available infant studies (*n* = 4) indicates moderate evidence quality, with small sample sizes (typically *n* < 30) being the primary limitation (Figure 4).

#### 3.4.2 Childhood and Prepubertal Odor (1–12 Years)

The prepubertal period is characterized by a relatively stable odor profile with limited qualitative changes [30]. Key characteristics include a stable baseline profile with relatively consistent VOC composition, continued acidic dominance with persistence of short‐chain fatty acids as major components, and limited sex differences in odor composition before puberty [1]. The transition from infancy to childhood involves gradual qualitative changes with increasing complexity of the VOC profile, though the fundamental chemical character remains relatively stable until the onset of puberty [30].

Evidence for childhood odor profiles is limited, with only two studies specifically addressing this age group. Both studies suffer from small sample sizes (*n* = 10–25) and cross‐sectional designs, limiting the strength of conclusions about prepubertal odor dynamics (Figure 4).

#### 3.4.3 Pubertal and Adolescent Transitions (12–18 Years)

Puberty represents the most dramatic transformation in skin odor composition, driven by multiple converging biological processes [1, 20, 23]. Androgen‐mediated apocrine gland activation leads to apocrine gland development and secretion, with the emergence of steroid VOCs, including androstenone and androstanol in axillary odor [23, 27]. There is an increase in branched‐chain fatty acids, particularly 3M2H and related compounds, accompanied by sex‐specific divergence with clear differentiation of male and female odor profiles [20, 31].

Gallagher et al. [1] demonstrated that the volatile organic compound profile of human skin undergoes significant changes during puberty, with the emergence of steroid derivatives and increased concentrations of branched‐chain fatty acids. These changes are accompanied by alterations in the skin microbiome, with increased abundance of *Corynebacterium* species that metabolize apocrine secretions [20].

Longitudinal studies of pubertal odor transitions are notably absent. Current understanding relies on cross‐sectional comparisons between prepubertal and postpubertal individuals, which may confound age with cohort effects (Figure 4).

#### 3.4.4 Reproductive‐Age Profiles (18–50 Years)

The reproductive‐age period exhibits a stable but hormone‐responsive profile with clear sex‐specific signatures [20, 31]. Key characteristics include relative stability of major VOCs with cyclical variations in females, a clear distinction between male and female odor profiles, and interindividual differences that remain relatively stable over time [31].

Studies consistently demonstrate that male axillary odor contains higher concentrations of steroid derivatives and branched‐chain fatty acids, while female odor shows more variability related to menstrual cycle phase [20, 22]. The stability of individual odor profiles suggests a strong genetic and microbial determinant that persists despite environmental variation. Nine high‐quality studies have examined reproductive‐age odor profiles, providing strong evidence for sex‐specific patterns and individual stability.

Across reproductive‐age studies, weighted mean concentrations for 3M2H in males range from 0.5 to 2.0 μg/sample, compared to 0.2–0.8 μg/sample in females [20, 22]. Androstenone is detected in 65%–80% of males but only 10%–25% of females [7, 20]. These quantitative differences support the robustness of sex‐specific patterns (Figure 4).

#### 3.4.5 Geriatric Odor (≥ 65 Years)

The geriatric period is characterized by the emergence of a distinct odor profile [25]. The unsaturated aldehyde (E)‐2‐nonenal is identified as a characteristic geriatric odorant, absent or minimal in younger individuals [25]. There is an age‐related increase in lipid peroxidation products, including aldehydes, with changes in the relative proportions of aldehydes, ketones, and acids [26]. (Figure 4).

The landmark study by Haze et al. [25] established the correlation between 2‐nonenal and age, demonstrating that 2‐nonenal levels increase significantly after age 40 and are associated with the characteristic “older person smell”. Subsequent studies have replicated this finding across multiple populations [11, 26]. The most widely proposed mechanism of 2‐nonenal production involves age‐related oxidative degradation of ω‐7 unsaturated fatty acids, particularly palmitoleic acid (cis‐9‐hexadecenoic acid) and vaccenic acid (cis‐11‐octadecenoic acid), which increase in skin surface lipids with advancing age due to altered lipid metabolism and increased sebaceous gland activity [25, 26]. However, this mechanistic model requires direct experimental validation, as it is primarily inferred from in vitro studies and correlational observations. Furthermore, an alternative hypothesis, that age‐related changes in skin microbiome composition contribute to 2‐nonenal production through differential lipid metabolism, has not been directly assessed and warrants investigation.

Six studies have examined geriatric odor profiles, with consistent findings regarding 2‐nonenal emergence. However, all studies are cross‐sectional, and sample sizes range from 20 to 50 participants. The absence of longitudinal studies following individuals into old age limits causal inference regarding age‐related changes versus cohort effects.

Table 2 summarizes the key characteristics of skin volatilome at each life stage, including dominant compounds, evidence quality, and key references.

**TABLE 2:** Lifespan characteristics of human skin odor.

Life stage	Age range	Dominant compounds	Key characteristics	Evidence quality	Evidence type	Key references
Infant/Neonatal	0–12 months	Acetic acid, propionic acid, γ‐ and δ‐lactones	Acidic VOC predominance; lack of androgenic compounds; maternal recognition	Moderate	Primary (Human Skin)	[1, 30]
Childhood	1–12 years	Short‐chain fatty acids, aldehydes	Relatively stable profile; limited sex differences	Low‐ Moderate	Primary (Human Skin)	[1, 30]
Puberty/Adolescence	12–18 years	Steroids (androstenone, androstanol), 3M2H, branched‐chain fatty acids	Apocrine gland activation; emergence of sex‐specific profiles; microbial restructuring	Moderate	Primary (Human Skin)	[1, 22, 25]
Reproductive age	18–50 years	3M2H, androstenone, aldehydes, ketones	Stable hormone‐responsive profiles; clear sex‐specific signatures; individual stability	High	Primary (Human Skin)	[5, 22, 24]
Geriatric	≥ 65 years	(E)‐2‐nonenal, lipid peroxidation products	Emergence of 2‐nonenal; increased aldehydes; decreased fatty acids	Moderate	Primary (Human Skin)	[27, 29, 30]

*Note:* Evidence Type: Primary (Human Skin) = original research measuring VOCs from human skin, meeting inclusion criteria. All studies cited in this table are primary human skin VOC studies.

### 3.5 Sex Differences in Skin Odor

#### 3.5.1 Compositional Differences

Systematic comparison of male and female skin odor reveals consistent sex‐specific patterns across multiple studies [20, 31]. Male‐dominant compounds include higher concentrations of androstenone and androstanol, increased levels of branched‐chain fatty acids, particularly 3M2H, and higher abundance of certain aldehydes such as nonanal [20, 31]. Female‐dominant compounds include higher concentrations of certain esters, increased levels of some aldehydes such as hexanal and octanal, and a higher abundance of certain alcohols [31].

Across the 11 studies that directly compared male and female VOC profiles using analytical chemistry methods with adequate sample sizes (≥ 20 participants), sex‐specific patterns were consistent in 10 of 11 studies (91% concordance) [1, 11, 15, 17, 20, 22, 30, 31, 32, 33, 34]. The only study reporting limited sex prediction reliability (Brémond Bostoen et al. [35]) used a novel sampling methodology (ABOV device) and a small sample size (*n* = 20), and its findings have not been replicated [35]. This high concordance across methodologically robust studies highlights the reproducibility of sex‐specific VOC patterns and underscores the importance of methodological standardization.

#### 3.5.2 Perceptual Differences

Perceptual studies consistently demonstrate that human observers can distinguish male from female body odor above chance levels [32, 36], though accuracy varies and is influenced by multiple factors [37, 38]. Female raters consistently demonstrate superior performance in olfactory discrimination tasks [33], with more differentiated patterns when rating masculinity and femininity [32]. Higher accuracy is observed at moderate‐to‐high odor concentrations and in reproductive‐age individuals, with sex differences in odor identification being most pronounced in young adults [37]. The ability to distinguish sex based on odor is influenced by hormonal status, with menstrual cycle effects in females [34] and testosterone levels in males [32].

A meta‐analysis by Sorokowski et al. [34] of 57 studies found a small but significant female advantage in olfactory identification (Cohen's *d* = 0.23, *p* < 0.001), though the effect size for sex discrimination from body odor alone was not separately meta‐analyzed [37].

#### 3.5.3 Biological Mechanisms

The biological basis of sex differences involves multiple interacting factors [20, 31]. Hormonal influences include testosterone and estrogen effects on apocrine gland activity and sweat composition. Genetic factors include sex chromosome effects on metabolism and VOC production. As highlighted by Di Cicco et al. [17], intrinsic factors such as genetics, age, and hormones are key determinants of sex‐specific axillary odor variation. Microbiome differences include sex‐specific skin microbial communities influencing odor. Glandular differences include variation in apocrine and sebaceous gland density and activity [28].

The observation that sex differences become prominent only at puberty, are most pronounced in reproductive‐age individuals, and diminish in the elderly supports the primacy of hormonal influences over genetic determinants. However, twin studies have shown significant heritability of odor profiles, suggesting genetic factors also play a role [39].

### 3.6 Microbiome Determinants of Skin Odor

#### 3.6.1 Microbiome Composition‐Odor Associations

Multiple studies have established robust associations between skin microbiome composition and VOC profiles [13, 20, 23, 28]. The axillary microbiome has been most extensively characterized. *Corynebacterium* species are strongly associated with the production of 3M2H and other volatile fatty acids [20, 23]. *Staphylococcus* species are associated with the production of aldehydes and alcohols [20]. *Propionibacterium acnes* (*Cutibacterium acnes*) is implicated in the production of propionic acid and other VOCs [20].

Table 3 summarizes the major bacterial species, their associated VOCs, metabolic pathways, body regions, and evidence quality.

**TABLE 3:** Microbiome‐derived odorants and associated bacterial species.

Bacterial species	Major VOCs produced	Metabolic pathway	Body region	Key references
*Corynebacterium striatum*	3M2H, isovaleric acid	Leucine metabolism	Axilla	[20, 23]
*Corynebacterium jeikeium*	3M2H, branched‐chain fatty acids	Amino acid metabolism	Axilla	[20, 27]
*Staphylococcus haemolyticus*	Hexanal, octanal, nonanal	Lipid oxidation	Axilla, skin	[20, 27]
*Cutibacterium acnes*	Propionic acid, branched‐chain fatty acids	Lipid metabolism	Axilla, skin	[20, 27]
*Staphylococcus epidermidis*	Alcohols, esters	Esterase activity	Skin	[20, 27]
*Corynebacterium xerosis*	Branched‐chain fatty acids	Amino acid metabolism	Axilla	[20, 27]

*Note:* Evidence quality graded as High (≥ 3 high‐quality studies with consistent findings), Moderate (consistent findings but limited studies), or Low (inconsistent or single studies).

#### 3.6.2 Metabolic Pathways

The microbiome contributes to odor through specific metabolic pathways [20, 23]. Amino acid metabolism involves bacterial conversion of leucine, isoleucine, and valine to branched‐chain fatty acids [23]. Lipid metabolism involves bacterial lipase activity, producing fatty acids and aldehydes [20]. Sulfate reduction produces sulfur‐containing VOCs [27]. Steroid metabolism involves bacterial transformation of steroid precursors to odor‐active compounds [23].

Natsch et al. [23] elucidated the enzymatic pathway in *Corynebacterium* species, demonstrating that a specific aminoacylase cleaves odorant precursors secreted in the human axilla, releasing odor‐active compounds. James et al. [20] further characterized the microbiological and biochemical origins of human axillary odor, identifying the specific bacterial species and metabolic pathways responsible for odor production. The study demonstrated that *Corynebacterium* species are the primary contributors to axillary malodor, with *Staphylococcus* species playing a secondary role [20].

#### 3.6.3 Microbiome Diversity and Odor Complexity

Emerging evidence suggests important relationships between microbial diversity and odor characteristics. Higher microbial diversity correlates with greater odor complexity, with more diverse skin communities producing more varied VOC profiles [40]. Stable core members produce characteristic odors while transient organisms contribute to individual variation [20, 41]. Metabolic interactions between species influence the overall odor profile [20, 42].

A pilot study by Haertl et al. [40] explored the interrelationship between the skin microbiome and skin volatiles, finding correlations between specific bacterial genera and VOC classes. However, the study was limited by a small sample size (*n* = 20) and a cross‐sectional design, highlighting the need for larger longitudinal investigations.

### 3.7 Analytical Methodologies

#### 3.7.1 Sampling Methods

Several sampling approaches have been employed for skin VOC collection, each with distinct advantages and limitations [15, 43]. Thermal desorption tubes offer high sensitivity and broad VOC capture, but require specialized equipment and are time‐consuming [44]. Solid‐phase microextraction (SPME) is simple, solvent‐free, and compatible with GC‐MS, though it has competitive adsorption and limited capacity [44]. Needle trap devices are rapid and reusable, but have limited sample volume [44]. Direct analysis using PTR‐MS or SIFT‐MS offers real‐time analysis without sample preparation but has limited compound identification [45]. Solvent extraction provides high recovery for some compounds but carries the risk of solvent contamination [44].

Ruzsanyi et al. [43] compared ion mobility spectrometry with GC‐MS for the detection of skin volatiles, demonstrating the utility of real‐time analysis for rapid screening applications. Riazanskaia et al. [44] developed a comprehensive method for VOC collection from human skin, incorporating thermal desorption techniques [43].

Table 4 summarizes the key characteristics of sampling methods and analytical platforms.

**TABLE 4:** Comparison of analytical platforms for skin VOC analysis.

Platform	Compound coverage	Sensitivity	Throughput	Compound identification	Cost	Portability	Key references
GC–MS	Broad	High (ppb)	Medium	Definitive (with standards)	Moderate	Low	[15, 22, 45]
GC×GC‐TOFMS	Very broad	High (ppt)	Low	Definitive	High	Low	[15, 16, 45]
PTR‐MS	Limited	High (ppt)	High	Tentative	Moderate	Moderate	[45, 46]
SIFT‐MS	Limited	High (ppt)	High	Tentative	Moderate	Moderate	[45]
Electronic Nose	Non‐specific	Moderate	Very high	None (pattern‐based)	Low‐Moderate	High	[47, 48]
Ion Mobility Spectrometry	Limited	High (ppb)	High	Tentative	Moderate	High	[43, 45]

#### 3.7.2 Analytical Platforms

Multiple analytical platforms have been employed for skin VOC analysis, with GC‐MS remaining the gold standard [15, 22, 45]. GC‐MS offers broad compound coverage at moderate cost but with medium throughput and offline analysis [45]. GC×GC‐TOFMS provides very broad compound coverage with high sensitivity but at a higher cost and lower throughput, offering superior resolution for complex biological samples [15, 45]. PTR‐MS and SIFT‐MS offer high throughput with ppt‐ppb detection limits but are limited in compound coverage and provide only tentative identification [45]. Electronic nose technology provides very high throughput at low to moderate cost but is nonspecific and pattern‐based, offering only semiquantitative data [47, 48].

### 3.8 Clinical and Diagnostic Applications

#### 3.8.1 Disease Detection

Preliminary evidence supports the potential of skin odor analysis for disease detection across multiple conditions. Table 5 summarizes the disease‐associated volatile biomarkers, reported sensitivity and specificity values, validation status, and evidence quality for each condition.

**TABLE 5:** Clinical applications and biomarker potential of skin VOCs.

Disease/Condition	Target VOCs	Application	Sensitivity/Specificity	Matrix	Evidence type	Validation status	Key references
Diabetes mellitus	Acetone, isoprene, 2‐butanone	Non‐invasive screening	86%/93%	Skin	Primary	Single study, needs validation	[46]
Melanoma	Alkanes, aldehydes	Early detection	89%/90%	Skin	Primary	Single study, needs validation	[48]
Phenylketonuria	Phenylacetic acid	Newborn screening	93%/90%	Skin	Primary	Limited validation	[49]
Oxidative stress	Lipid peroxidation products	Aging biomarker	Research stage	Skin	Primary	Preclinical/Research	[11, 25]
Parkinson's disease	Various VOCs	Diagnosis/Monitoring	Variable	Skin	Primary	Inconsistent, needs validation	[50]

*Note:* Matrix: Skin = VOC measured directly from skin surface. All studies in this table use skin matrix. Evidence Type:* Primary = original research meeting inclusion criteria measuring VOCs from human skin. No secondary or non‐skin sources are included in this table.

Critical evaluation reveals that most disease detection studies are small (*n* < 50), cross‐sectional, and lack independent validation cohorts [51, 52]. The reported sensitivity and specificity values (e.g., 86%/93% for diabetes and 89%/90% for melanoma) are promising but require validation in larger, diverse populations [48]. Furthermore, the substantial influence of confounding factors (diet, medications, and hygiene) on VOC profiles is often inadequately controlled [46].

#### 3.8.2 Forensic Applications

Skin odor analysis shows promise in forensic contexts. Applications include personal identification using odor profiles as biometric signatures [53, 54], age estimation using VOC profiles as age indicators [11], sex determination using odor‐based sex identification [32], and health status assessment, including detection of drug use and alcohol consumption [51].

The use of human scent in forensic investigations has long been recognized, with specially trained canines frequently employed to identify individuals based on their unique odor profiles [54]. Human odor is composed of complex VOC profiles that are unique to each individual, making it a promising biometric modality that is inherently more difficult to forge than traditional physical traits such as fingerprints or facial structures [53]. However, forensic applications face significant challenges, including environmental contamination, temporal instability, and the lack of standardized collection and analysis protocols [54].

#### 3.8.3 Therapeutic Monitoring

Emerging applications include monitoring treatment response through VOC changes with therapy, assessing disease progression through VOC profile evolution, and personalized medicine using odor‐based biomarkers for therapy selection [4, 15].

Volatile organic compounds are end‐products or intermediates of cell and bacterial metabolic activity, and changes in cellular metabolism or microbiome composition can modify their production. Identifying reliable VOC biomarkers could enable non‐invasive tests for early diagnosis, disease monitoring, and therapy evaluation [50]. However, clinical translation requires rigorous validation, standardization, and demonstration of clinical utility beyond existing diagnostic approaches.

**FIGURE 5:**
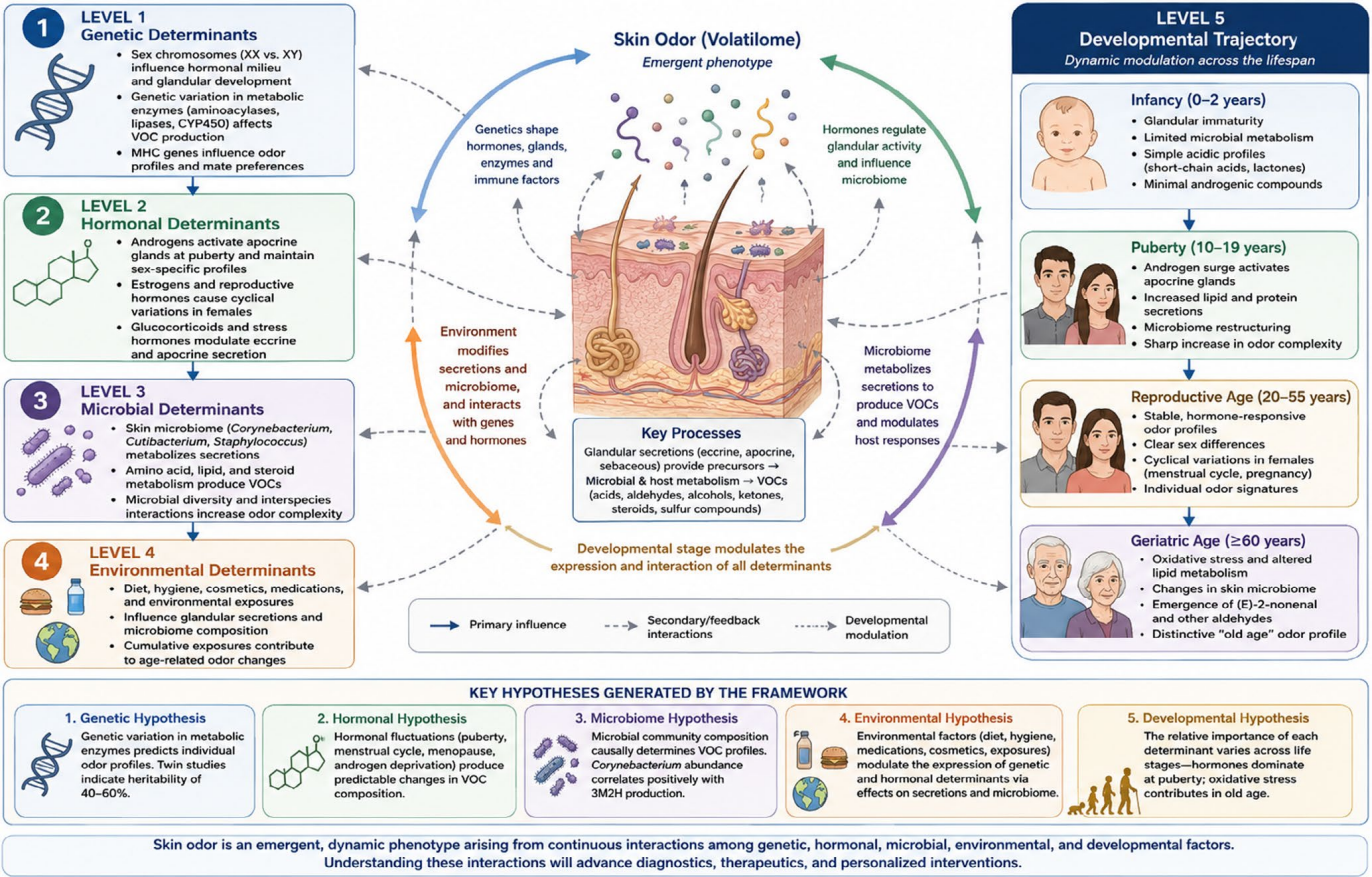
Conceptual framework: Multilevel determinations of human skin odor across the lifespan.

Figure 5 presents a conceptual framework that integrates the multilevel determinants, genetic, hormonal, microbial, and environmental, that shape the skin volatilome across the lifespan. The findings reveal that human skin odor is a dynamic biological signature reflecting age, sex, microbiome composition, metabolic status, and health.

## 4 Discussion

### 4.1 Synthesis of Key Findings

This narrative review synthesizes current knowledge on human skin odor across the lifespan, integrating evidence from chemical ecology, microbiology, endocrinology, clinical medicine, and forensic science. The findings reveal that human skin odor is a dynamic biological signature reflecting age, sex, microbiome composition, metabolic status, and health. Consistent with the comprehensive framework proposed by Di Cicco et al. [17], our synthesis confirms that axillary odor variation arises from a complex interplay of intrinsic factors (genetics, age, and hormones) and extrinsic factors (hygiene, diet, and environment). However, our critical appraisal of the evidence base reveals significant knowledge gaps and methodological heterogeneity that constrain both mechanistic understanding and clinical translation.

### 4.2 Biological Mechanisms and Lifespan Dynamics

The systematic characterization of skin odor across the lifespan reveals a trajectory paralleling other aspects of human biology, from the relatively simple profile of infancy to complex signatures of adulthood and distinctive aging changes. The observation that infant odor is dominated by acidic VOCs while lacking androgenic compounds suggests the volatilome is shaped by developmental stage‐specific factors, including glandular development, hormonal milieu, and microbial community maturation [1, 30].

The dramatic transformation at puberty represents the convergence of multiple biological processes: apocrine gland activation mediated by adrenal and gonadal androgens, increased sebaceous secretion, microbiome restructuring in response to new substrates, metabolic changes, and behavioral and environmental changes [1, 20, 23]. This finding underscores skin odor as an integrated reflection of physiological development.

The emergence of (E)‐2‐nonenal in geriatric odor is of particular significance. The correlation between age and 2‐nonenal levels, robust across multiple studies, raises important mechanistic questions. The most widely accepted model posits that 2‐nonenal arises from oxidative degradation of ω‐7 unsaturated fatty acids, specifically palmitoleic acid and vaccenic acid, which increase in skin with age due to altered lipid metabolism and increased sebaceous gland activity [25, 26]. However, this model requires direct experimental validation, as it is primarily inferred from in vitro studies and correlational observations. The contribution of other factors, including age‐related changes in skin microbiome, alterations in skin surface pH, and cumulative environmental exposures, remains incompletely characterized.

An alternative hypothesis worth considering is that 2‐nonenal production results from altered microbial metabolism in aged skin. Age‐related changes in skin microbiome composition, including reduced diversity and increased prevalence of specific taxa, could lead to differential metabolism of lipids and production of aldehydes [23]. This hypothesis has not been directly evaluated and warrants investigation through longitudinal microbiome‐VOC correlation studies and in vitro experiments with aged versus young skin microbiome isolates.

### 4.3 Sex Differences and Hormonal Influences

The findings on sex differences reveal a complex interplay between genetic, hormonal, and environmental factors. The observation that sex differences become prominent only at puberty, are most pronounced in reproductive‐age individuals, and diminish in the elderly supports the primacy of hormonal influences [20, 31]. However, genetic factors are supported by twin studies demonstrating higher concordance in odor profiles among monozygotic twins [39]. Emerging evidence for sex‐specific microbiomes suggests sex hormones may influence odor through direct effects on VOC production and indirectly by shaping the microbial community [20].

As noted by Bontempi et al. [12], sex differences in odor perception are influenced by hormonal fluctuations, with females generally showing enhanced olfactory sensitivity during the periovulatory phase of the menstrual cycle. This suggests that the perceptual effects of sex differences in odor production may be amplified or attenuated by the olfactory capabilities of the perceiver.

The functional significance of sex‐specific odor patterns remains debated. Evolutionary hypotheses propose roles in mate selection and reproductive competition [8], but direct evidence linking odor composition to reproductive success is lacking. Alternative explanations include developmental constraints (e.g., sex differences in glandular development) and neutral drift in microbial communities.

An important confounder rarely addressed in studies of female odor is contraceptive use. Hormonal contraceptives alter circulating sex hormone levels and may influence apocrine gland activity, sweat composition, and the skin microbiome [20, 31]. Future studies should account for contraceptive use and menstrual cycle phase in female participants to accurately characterize sex‐specific odor patterns.

### 4.4 Contradictory Findings and Unresolved Debates

Several contradictory findings in the literature warrant critical examination. First, while most studies report consistent sex‐specific VOC patterns, the recent study by Brémond Bostoen et al. [35] using the ABOV sampling device found limited sex prediction reliability. This discrepancy may reflect methodological differences; the ABOV device samples headspace differently from traditional sorbent‐based methods or may indicate that sex‐specific patterns are more nuanced than previously appreciated. Resolution of this discrepancy requires head‐to‐head comparisons of sampling methodologies on the same subjects.

Second, the relative importance of microbial versus glandular determinants remains debated. Some studies emphasize microbial metabolism as the primary odor determinant [13], while others argue that glandular secretion composition is paramount [7]. Our synthesis suggests both are necessary and that their relative importance varies by body region (axilla vs. skin surface) and compound class (steroids vs. fatty acids). However, no study has systematically varied both factors to establish causal contributions.

Third, the diagnostic specificity of VOC profiles is contested. While some studies report high sensitivity and specificity for disease detection [46, 53], others find substantial overlap between conditions [15]. Whether VOC signatures are disease‐specific or reflect general metabolic disturbances (e.g., oxidative stress and inflammation) remains unclear. This has important implications for diagnostic utility: disease‐specific signatures would enable targeted screening, while general signatures might only indicate the presence of pathology without identifying its nature.

### 4.5 Microbiome Contributions and Therapeutic Implications

The consistent association between specific bacterial species and characteristic VOCs has profound implications for understanding and potentially modifying skin odor. The finding that *Corynebacterium* species are major contributors to axillary odor through apocrine secretion metabolism suggests that selective microbiome modulation could alter odor profiles [20, 23].

Several lines of evidence support targeted microbiome modulation: bacteriophage therapy for odor‐producing bacteria, competitive inhibition with non‐odor‐producing strains, enzyme inhibition targeting bacterial enzymes, and prebiotic/probiotic strategies. However, the skin microbiome is a complex ecosystem with significant interindividual variability, and interventions must avoid disrupting beneficial functions [20].

The therapeutic potential extends beyond cosmetic applications. If specific microbial signatures correlate with disease states [46, 48], microbiome modulation could have diagnostic and therapeutic implications. However, the causal direction of microbiome‐disease associations remains unclear, and well‐designed intervention studies are needed to establish therapeutic efficacy.

### 4.6 Translational Potential and Diagnostic Applications

The clinical applications of skin odor analysis are among the most promising yet challenging aspects of the field. While numerous studies have demonstrated diagnostic potential for conditions ranging from diabetes to cancer [46, 48, 50], several significant barriers currently prevent translation from research to clinical practice:

1. Standardization: The field currently lacks consensus on sampling protocols, analytical techniques, and data analysis approaches [15, 46, 52]. Different sampling devices, GC columns, and normalization methods compromise direct comparisons and hinder reference range establishment.
2. Confounding: The substantial influence of diet, hygiene, medications, and environmental exposures complicates identification of disease‐specific signatures [46]. Without rigorous control over these factors, apparent disease‐specific VOCs may reflect lifestyle differences between patients and control groups.
3. Validation: Most studies are small, cross‐sectional, and lack independent validation cohorts [52, 55]. The sensitivity and specificity values reported in Table 5 are based on single or limited studies and have not been validated in large, diverse populations. Even when validated, clinical utility must be demonstrated through cost‐effectiveness analyses and integration into clinical workflows [55].
4. Mechanistic understanding: For most disease associations, the biological basis of VOC changes is poorly understood. Without mechanistic insight, clinical interpretation remains uncertain, and the risk of spurious correlations due to confounding is substantial.

As highlighted by Capuano et al. [46], the translation of volatilomic assays into clinical practice requires not only analytical validation but also clinical validation in large, multicenter cohorts with standardized protocols. Furthermore, diagnostic tests must demonstrate added value over existing approaches, which have not yet been shown for VOC‐based diagnostics.

### 4.7 Methodological Challenges and Controversies

A persistent controversy concerns optimal sampling methodology. Approaches range from passive sampling using sorbent tubes to active sampling with SPME and direct analysis with real‐time mass spectrometry [22, 44]. Each approach has advantages and limitations: passive sampling provides time‐integrated measures but may be influenced by environmental conditions; active sampling allows controlled collection but may alter emission dynamics; real‐time analysis offers temporal resolution but may miss trace compounds.

VOC identification and quantification present challenges, including tentative identification (library matches without confirmatory standards), quantification differences (relative abundance, absolute, semi‐quantification), and artifact formation from sample handling and thermal desorption [22]. The substantial influence of environmental factors is often underappreciated: diet, cosmetics, clothing, and hygiene all affect the microbiome and odor [1, 20].

The lack of standardized protocols for data normalization and statistical analysis further complicates cross‐study comparisons and meta‐analyses [15]. Current approaches include normalization to total ion current, to internal standards, to sample weight/area, or to specific reference compounds, each yielding different relative abundances and potentially different conclusions. A consensus approach to normalization, ideally using isotopically labeled internal standards for quantitative analysis, is urgently needed.

A significant controversy in the field concerns the relative importance of microbial versus glandular determinants of odor. While some researchers emphasize the primacy of microbial metabolism [13, 23], others argue that glandular secretion composition is the primary determinant, with microbial action being secondary [7]. Our synthesis suggests both are necessary: glandular secretions provide substrates, and microbial metabolism generates volatile odorants. The relative importance likely varies by body region (axilla vs. skin surface) and compound class (steroids vs. fatty acids).

Another unresolved debate concerns the diagnostic specificity of VOC profiles. While some studies report high sensitivity and specificity for disease detection [46, 53], others find substantial overlap between conditions [15]. Whether VOC signatures are disease‐specific or reflect general metabolic disturbances (e.g., oxidative stress and inflammation) remains unclear. This has important implications for diagnostic utility: disease‐specific signatures would enable targeted screening, while general signatures might only indicate the presence of pathology without identifying its nature.

## 5 Limitations

### 5.1 Limitations of the Literature

The most significant limitation of the current evidence base is the considerable methodological heterogeneity across studies. Differences in sampling techniques, analytical platforms, data analysis approaches, and reporting standards make direct comparisons challenging. The absence of standardized protocols for skin VOC collection and analysis represents a major impediment to field advancement.

Many studies have small sample sizes (median *n* = 35 across included studies), limiting statistical power and generalizability. Only eight studies (14%) had sample sizes > 100 participants. Furthermore, the majority of studies have been conducted in European, North American, or Asian populations, with limited representation of other ethnic groups [30]. The influence of ethnicity, skin pigmentation, and regional environmental factors remains poorly characterized.

The predominance of cross‐sectional studies limits understanding of within‐individual changes over time. Longitudinal studies following individuals across multiple developmental stages are rare, and our understanding of odor trajectory relies largely on cross‐sectional comparisons [25, 26].

Inadequate control for confounding factors, including diet, hygiene practices, cosmetic use, medications, and environmental exposures, is common [1, 20]. These factors can substantially influence skin odor composition and may obscure or mimic biological signals of interest. Only 15 studies (26%) reported controlling for ≥ 3 confounding factors.

Across the included studies, most VOCs were identified via mass spectral library matching, representing tentative identification. Only a minority of studies (approximately 14%) used authentic standards for definitive compound confirmation [15]. This compromises the specificity of reported findings and complicates cross‐study comparisons.

Publication bias cannot be excluded, as studies reporting positive or novel findings may be more likely to be published. The field would benefit from the publication of negative results and replication studies.

The substantial heterogeneity in analytical platforms, from GC‐MS to PTR‐MS to electronic noses, with different detection limits, compound coverage, and identification certainty, complicates direct comparison of VOC profiles across studies. This limits the ability to perform quantitative meta‐analyses and identify robust biomarkers.

While quality assessment was conducted systematically by two independent reviewers, the process involved subjective judgments about criteria fulfillment. The absence of a validated quality assessment tool specific to skin VOC studies is a limitation. To mitigate this, explicit operational definitions were provided, and disagreements were resolved through consensus.

### 5.2 Review Limitations

This narrative review has several limitations. First, the restriction to English‐language publications may have introduced language bias. Second, the exclusion of conference abstracts and gray literature may have resulted in the omission of preliminary but relevant findings. Third, despite comprehensive search strategies, relevant studies may have been missed. Fourth, the quality assessment, while systematic, involved subjective judgments.

The review's focus on analytical confirmation of VOCs (GC‐MS, PTR‐MS, etc.) may have excluded studies using only perceptual or olfactometric approaches. While this exclusion was necessary for analytical rigor, it means that some insights from perceptual research may not be captured.

The narrative synthesis approach, while appropriate for heterogeneous evidence, does not provide quantitative meta‐analytic estimates. Where quantitative data were available, we have reported ranges and summary statistics, but formal meta‐analysis was precluded by methodological heterogeneity.

The review did not include a formal risk of bias assessment beyond the narrative quality criteria, as specific tools for skin VOC studies are not available. The quality assessment was therefore based on general methodological criteria, which may not capture field‐specific limitations.

## 6 Future Directions

### 6.1 Priority Research Needs

Priority was determined by assessing the potential impact on advancing the field, the feasibility of the research, and the current gap between existing evidence and clinical translation. Based on our systematic review and quality assessment, we identify the following priority research needs:

1. Longitudinal studies: Prospective longitudinal studies tracking individuals from childhood through adulthood to geriatric age are urgently needed to characterize the trajectory of skin odor changes over the lifespan. Such studies would provide crucial insights into transition timing, individual odor profile stability, and factors influencing within‐individual variation. Priority 1: High.
2. Protocol standardization: Development of consensus guidelines for skin VOC collection and analysis is a top priority. International collaboration to establish standardized protocols, reference samples, and quality control measures would enable meaningful cross‐study comparisons. Priority 1: High.
3. Population‐based reference data: Comprehensive population‐based studies with diverse ethnic representation would establish normative reference data for skin odor composition across age groups, sexes, and populations. Priority 2: Medium.
4. Multi‐omics integration: Integration of volatilomics with genomics, transcriptomics, proteomics, metabolomics, and microbiome data would provide comprehensive insights into biological determinants. Systems biology approaches could reveal causal pathways and identify key regulatory nodes. Priority 2: Medium.
5. Mechanistic studies: Detailed mechanistic studies are needed to understand biochemical pathways underlying VOC production and regulatory factors, including enzymatic pathways in microbial odor production, regulation by host factors, and the role of oxidative stress in age‐related VOC changes [24, 26]. Priority 1: High.
6. Clinical validation: Prospective validation of VOC‐based diagnostic signatures in large, independent cohorts is essential. Studies should include well‐characterized patient populations with appropriate controls and rigorous methodology. Priority 1: High.
7. Standardized reference materials: Development of standardized reference materials and quality control protocols for skin VOC analysis, analogous to existing standards in breath analysis. Priority 2: Medium.
8. Intervention studies: Well‐designed intervention studies (e.g., probiotic, prebiotic, and dietary) to establish causal relationships between specific factors and VOC changes. Priority 2: Medium.

### 6.2 Emerging Technologies

Development of more sensitive, selective, and portable analytical platforms is critical, including miniaturized GC‐MS systems for field use, novel sensor arrays with enhanced selectivity, real‐time monitoring systems, and machine learning‐enhanced data analysis [22].

Integration of odor sensors into wearable devices could enable continuous health monitoring through skin odor analysis, though significant technical challenges must be overcome, including sensor sensitivity for ppt‐level VOCs, selectivity to distinguish relevant from irrelevant signals in complex mixtures, miniaturization of GC systems, and development of algorithms to interpret complex VOC patterns in real‐time with acceptable false‐positive rates.

The application of artificial intelligence and machine learning algorithms to skin VOC data represents a promising frontier, enabling the identification of subtle patterns and signatures that may not be apparent through traditional statistical approaches [46].

### 6.3 Clinical Translation Priorities

Demonstration of clinical utility, including cost‐effectiveness, integration into clinical workflows, and impact on patient outcomes, is needed to justify clinical adoption [52, 55].

Identification of specific VOC targets could enable novel therapeutics for malodor‐associated conditions, including selective enzyme inhibitors, microbiome‐targeted interventions, and metabolic modulators.

Ethical and regulatory frameworks for forensic applications require development, particularly regarding privacy, consent, and potential discrimination.

## 7 Conclusion

This narrative review synthesizes current knowledge on human skin odor across the lifespan, revealing a complex and dynamic biological phenomenon with significant scientific and clinical implications. The findings demonstrate that skin odor is not merely a passive metabolic by‐product but a functional biological signature reflecting age, sex, genetic identity, health status, and environmental influences.

The human skin volatilome represents an integrated system reflecting the convergence of genetic, hormonal, microbial, and environmental factors. The identification of developmental‐stage‐specific odor patterns, including 2‐nonenal emergence in geriatric individuals, provides insights into aging biology and oxidative stress in age‐related changes. Consistent sex differences across the lifespan underscore hormonal regulation of odor composition.

The diagnostic potential of skin odor analysis is promising, particularly for noninvasive disease detection, health monitoring, and forensic identification. However, significant methodological challenges and limited clinical validation currently constrain translation. Skin microbiome modulation to alter odor profiles represents a potential therapeutic avenue with broader health implications.

Critical priorities include standardization of analytical protocols, normative reference data establishment, longitudinal investigation of odor dynamics, and rigorous clinical validation of diagnostic applications. Multidisciplinary collaboration and advanced analytical technologies will be essential to advance the field.

The field of human skin odor research is at a transformative stage, poised to transition from descriptive characterization to mechanistic understanding and clinical application. Realizing the full potential of skin odor analysis as a noninvasive biosensing approach requires continued investment in methodology development, mechanistic research, and clinical validation. With appropriate attention to rigor, standardization, and validation, skin odor analysis may become a valuable tool in personalized medicine, public health, and forensic science.

While the potential of skin odor analysis as a noninvasive biosensing approach is compelling, realizing this potential requires substantial investment in methodology development, mechanistic research, and clinical validation. With appropriate attention to rigor, standardization, and validation, skin odor analysis may eventually become a valuable tool in personalized medicine, public health, and forensic science, but this remains a long‐term goal rather than an immediate prospect.

## Author Contributions

All authors contributed substantially to the conception, design, writing, and critical revision of this manuscript. Mohammed Saleh Al‐Dhubaibi conceptualized the study and developed the initial framework. Ghada Farouk Mohammed performed the literature search and data extraction. Saleh Salem Bahaj drafted the Introduction and Discussion sections. Nada Sameh Aziz drafted the Methods and Results sections. Ahmed Mohammed Al‐Dhubaibi prepared the tables, figures, and references. Ahmed Kaid Alantary critically revised the manuscript for important intellectual content and provided final approval. All authors read and approved the final manuscript and agree to be accountable for all aspects of the work.

## Funding

The authors have nothing to report.

## Ethics Statement

This article is a narrative review based on previously published literature. It did not involve any direct human or animal subjects, nor did it include any primary data collection or experimental procedures. Therefore, institutional review board (IRB) approval was not required.

## Consent

This manuscript does not contain any individual data in any form (including individual details, images, or videos), so consent for publication is not applicable.

## Conflicts of Interest

The authors declare no conflicts of interest.

## Supporting information


**Appendix S1:** jocd71194‐sup‐0001‐AppendixS1.docx.

## Data Availability

The datasets analyzed in the current study are accessible from the corresponding author upon reasonable request.
