# Supplementary material for: Human Skin Odor Throughout Life: A Literature Review
Source: J Cosmet Dermatol. 2026 Sep 15;25(9):e71194. doi: 10.1111/jocd.71194 (PMC13577823; doi:10.1111/jocd.71194)
Supplement: Supplementary file 1 [file JOCD-25-e71194-s001.docx]

**Appendix A: Complete Database Search Strategies**

**A.1 PubMed Search Strategy**

("skin odor"[tiab] OR "body odor"[tiab] OR "axillary odor"[tiab] OR "skin volatilome"[tiab] OR "cutaneous volatile organic compounds"[tiab] OR "skin VOCs"[tiab] OR "human scent"[tiab])

AND

("microbiome"[tiab] OR "microbiota"[tiab] OR "Corynebacterium"[tiab] OR "Staphylococcus"[tiab] OR "Cutibacterium"[tiab] OR "Propionibacterium"[tiab])

AND

("age"[tiab] OR "aging"[tiab] OR "lifespan"[tiab] OR "infant"[tiab] OR "child"[tiab] OR "adolescent"[tiab] OR "adult"[tiab] OR "elderly"[tiab] OR "geriatric"[tiab])

AND

("sex differences"[tiab] OR "sex"[tiab] OR "male"[tiab] OR "female"[tiab] OR "gender"[tiab])

AND

("gas chromatography"[tiab] OR "mass spectrometry"[tiab] OR "electronic nose"[tiab] OR "PTR-MS"[tiab] OR "GC-MS"[tiab] OR "SPME"[tiab] OR "thermal desorption"[tiab])

*Search Date: May 15, 2026*

*Limits: English language*

**A.2 Scopus Search Strategy**

TITLE-ABS-KEY("skin odor" OR "body odor" OR "axillary odor" OR "skin volatilome" OR "cutaneous volatile organic compounds" OR "skin VOCs" OR "human scent")

AND TITLE-ABS-KEY("microbiome" OR "microbiota" OR "Corynebacterium" OR "Staphylococcus" OR "Cutibacterium" OR "Propionibacterium")

AND TITLE-ABS-KEY("age" OR "aging" OR "lifespan" OR "infant" OR "child" OR "adolescent" OR "adult" OR "elderly" OR "geriatric")

AND TITLE-ABS-KEY("sex differences" OR "sex" OR "male" OR "female" OR "gender")

AND TITLE-ABS-KEY("gas chromatography" OR "mass spectrometry" OR "electronic nose" OR "PTR-MS" OR "GC-MS" OR "SPME" OR "thermal desorption")

*Search Date: May 15, 2026*

*Limits: English language*

**A.3 Web of Science Search Strategy**

TS=("skin odor" OR "body odor" OR "axillary odor" OR "skin volatilome" OR "cutaneous volatile organic compounds" OR "skin VOCs" OR "human scent")

AND TS=("microbiome" OR "microbiota" OR "Corynebacterium" OR "Staphylococcus" OR "Cutibacterium" OR "Propionibacterium")

AND TS=("age" OR "aging" OR "lifespan" OR "infant" OR "child" OR "adolescent" OR "adult" OR "elderly" OR "geriatric")

AND TS=("sex differences" OR "sex" OR "male" OR "female" OR "gender")

AND TS=("gas chromatography" OR "mass spectrometry" OR "electronic nose" OR "PTR-MS" OR "GC-MS" OR "SPME" OR "thermal desorption")

*Search Date: May 15, 2026*
*Limits: English language*

**A.4 Additional Search Methods**

In addition to the database searches described above, the following supplementary search methods were employed:

1. **Reference list screening:** The reference lists of all included studies and relevant reviews were manually screened to identify additional potentially eligible studies not captured by the electronic database searches.
2. **Citation tracking:** Google Scholar was used to perform forward citation tracking on key publications to identify studies that had subsequently cited them.
3. **Hand searching:** Key journals in the field (e.g., *Journal of Investigative Dermatology*, *British Journal of Dermatology*, *Journal of Chemical Ecology*, *Analytical Chemistry*) were manually screened for relevant articles published within the last two years.
